# Digital PCR to Detect and Quantify Heteroresistance in Drug Resistant *Mycobacterium tuberculosis*

**DOI:** 10.1371/journal.pone.0057238

**Published:** 2013-02-27

**Authors:** Suporn Pholwat, Suzanne Stroup, Suporn Foongladda, Eric Houpt

**Affiliations:** 1 Division of Infectious Diseases and International Health, Department of Medicine, University of Virginia, Charlottesville, Virginia, United States of America; 2 Department of Microbiology, Faculty of Medicine Siriraj Hospital, Mahidol University, Bangkok, Thailand; National Institute of Allergy and Infectious Disease, United States of America

## Abstract

Drug resistance in *Mycobacterium tuberculosis* presents an enormous public health threat. It is typically defined as >1% of drug resistant colonies using the agar proportion method. Detecting small numbers of drug resistant Tb in a population, also known as heteroresistance, is challenging with current methodologies. Here we have utilized digital PCR to detect heteroresistance within *M*. *tuberculosis* populations with excellent accuracy versus the agar proportion method. We designed dual TaqMan-MGB probes to detect wild-type and mutant sequences of *katG* (315), *rpoB* (531), *gyrA* (94,95) and *rrs* (1401), genes that associate with resistance to isoniazid, rifampin, fluoroquinolone, and aminoglycoside respectively. We generated heteroresistant mixtures of susceptible and extensively drug resistant Tb, followed by DNA extraction and digital PCR. Digital PCR yielded a close approximation to agar proportion's percentages of resistant colonies, and yielded 100% concordance with agar proportion's susceptible/resistant results. Indeed, the digital PCR method was able to identify mutant sequence in mixtures containing as little as 1000∶1 susceptible:resistant Tb. By contrast, real-time PCR or PCR followed by Sanger sequencing were less sensitive and had little resolution to detect heteroresistance, requiring fully 1∶1 or 10∶1 susceptible:resistant ratios in order to detect resistance. Our assay can also work in sputum so long as sufficient quantities of Tb are present (>1000 cfu/ml). This work demonstrates the utility of digital PCR to detect and quantify heteroresistance in drug resistant Tb, which may be useful to inform treatment decisions faster than agar proportion.

## Introduction

Multidrug-resistant tuberculosis (MDR-TB), defined as resistance of >1% of colonies to isoniazid (INH) and rifampin (RIF), was found in 3.3% of new cases and 21% of recurrent TB cases globally [1], and many strains harbor extensive resistance to additional drugs. Some TB patients harbor mixed populations of drug susceptible and resistant organisms, a phenomenon which is referred to as heteroresistance [2].

Heteroresistance allows subpopulations of bacteria to grow despite the presence of antibiotics and is thus a precursor to full resistance [3]. The phenomenon is not rare in *M. tuberculosis* and has been reported for isoniazid, rifampin, ethambutol, streptomycin, and fluoroquinolones [4]–[6]. It can be detected by culture-based methods (i.e., when more than 1 but less than 100% of colonies are resistant to drug), or by simultaneous detection of wild-type and mutated sequences using PCR-based techniques, such as restriction fragment length polymorphism [4], sequencing [7] or line probe assays [6], [8].

Most of the latter PCR-based methods are laborious and technically difficult. Moreover, detection of low amounts of mutant template is challenging with PCR since the abundant (in this case wild-type) sequence may preferentially amplify. This possibility urges caution given the proliferation of PCR-based methods to detect MDR TB in the field. For example, it has been reported that between 65% and 100% of *rpoB* mutant DNA is required to be detected by the Xpert MTB/RIF assay [9]. Likewise, the line probe based Genotype MTBDR assay can detect heteroresistance of MTB but requires that the relative proportion of resistant organisms is ≥10% [8]. Recently, a molecular beacon assay was developed to detect mutant targets of *gyrA* gene, but again required the presence of 5–10% of mutant DNA to be detected in a mixture with wild type DNA [5]. Importantly, if detected, none of these assays yield a precise measure of how much heteroresistance exists.

In 1999 the term digital PCR was used to describe dilution of DNA to single copies to improve the sensitivity of PCR to detect rare cancer-associated mutations amidst an excess of wild-type sequence [10]. Here we have investigated the role of digital PCR to detect and quantify heteroresistance within mixed Tb populations.

## Materials and Methods

### Mycobacterial strains and culture conditions

Mycobacterial strains used in this study included *M. tuberculosis* H37Rv (ATCC 27294) and a clinical extensively drug resistant (XDR) TB isolate (100% resistant to isoniazid, rifampin, ofloxacin, amikacin, kanamycin, streptomycin by agar proportion method, with the *katG*315, *rpoB*531, *gyrA*94,95, and *rrs*1401 mutations) from the Mycobacteriology Service Unit, Department of Microbiology, Faculty of Medicine Siriraj Hospital, Mahidol University, Bangkok, Thailand. All work was approved by the University of Virginia Institutional Biosafety Committee and Human Investigation Committees. Tb isolates were cultured on Lowenstein-Jensen medium at 37°C for two weeks. Cell suspensions were prepared in Middlebrook 7H9 (M7H9) broth supplemented with Middlebrook OADC enrichment (Difco, Livonia, MI, USA) and adjusted to 0.5 McFarland.

### H37Rv:XDR-TB mixtures

The 0.5 McFarland XDR-TB was diluted 10-fold serially in M7H9 plus OADC. One ml of each XDR-TB suspension (undiluted, 1/10, 1/100, 1/1000) was mixed with 1 ml of 0.5 McFarland H37Rv to construct mixtures of 1∶1, 10∶1, 100∶1, and 1000∶1 respectively.

### DNA extraction

DNA was isolated from each TB mixture. Briefly, 1 ml of 0.5 McFarland H37Rv, XDR-TB and each H37Rv:XDR-TB mixtures were transferred to 2 ml screw cap tube, centrifuged at 21,000×g for 10 min, and the pellets were re-suspended in 1 ml Tris-EDTA (TE) buffer, boiled at 100°C for 30 min, centrifuged at 21,000×g for 3 min, and supernatants were stored at −20°C as DNA template.

### Oligonucleotides

Primers and probes for *katG, rpoB*, *gyrA*, and *rrs* were sourced based on sequences of H37Rv and the XDR-TB isolate. The *rpoB* primers and the mutant probe were those reported by Espasa et al [11], while the primers and probes of *katG* were those of Yesilkaya et al [12]. The primers and probes of *gyrA*, *rrs*, and the *rpoB* wild-type probe were designed for this work using Primer Express3 (Applied Biosystems, Life Technologies Corporation, Carlsbad, CA, USA). All sequences are shown in Table 1.

**Table 1:** Primer and probe sequences.

Gene	Primers and Probes	Sequences
*katG*	*katG*-F	5′-TGCTCCGCTGGAGCAGAT-3′
	*katG*-R	5′-TCGAGGAAACTGTTGTCCCATT-3′
	*katG*315-wt	5′-FAM-CGATGCCGCTGGT-MGB-3′
	*katG*315-mt	5′-VIC-CGATGCCGGTGGT-MGB-3′
*rpoB*	*rpoB*-F	5′-ACCGCAGACGTTGATCAACAT-3′
	*rpoB*-R	5′-GGCACGCTCACGTGACAG-3′
	*rpoB*531-wt	5′-TexaRed-CCCAGCGCCGACAGTCGG-3′
	*rpoB*531-mt	5′-Cy5-CAGCGCCAACAGTCGGCG-3′
*gyrA*	*gyrA*-F	5′-AGACCATGGGCAACTACCAC-3′
	*gyrA*-R	5′-TTTCCCTCAGCATCTCCATC-3′
	*gyrA*94,95-wt	5′-FAM-GATCTACGACAGCCTGG-MGB-3′
	*gyrA*94,95-mt	5′-VIC-GATCTACGGCACCCTG-MGB-3′
*rrs*	*rrs*-F	5′-AATCCTTAAAAGCCGGTCTCA-3′
	*rrs*-R	5′-CTCCCTCCCGAGGGTTAG-3′
	*rrs*1401-wt	5′-FAM-GCCCGTCACGTCAT-MGB-3′
	*rrs*1401-mt	5′-VIC-CCCGTCGCGTCAT-MGB-3′

### Real-Time PCR

PCR mixtures (10 µl) consisted of 5 µl of 2×Quantitect Multiplex NoROX PCR master mix (Qiagen Inc, Valencia, CA, USA), 0.1 µl of 50 µM of each forward and reverse primer except for *katG* (0.16 µl), 0.04 µl of 50 µM of each wild-type and mutant probe, 2.72 µl nuclease-free water except for *katG* (2.6 µl) and µl of DNA template. Real-time PCR of each gene was performed on a ViiA7 (Applied Biosystems, Life Technologies Corporation, Carlsbad, CA, USA) with initial denaturation at 95°C for 10 min, followed by 45 cycles denaturation at 95°C for 15 sec, and annealing and extension at 63°C for 1 min.

### Digital PCR

DNA from each H37Rv:XDR-TB mixture was diluted 10 fold serially in nuclease free water and subjected to real-time PCR for *rpoB* in 10 replicates in order to calculate the percentage of positive detections at each dilution. The 1/10000 dilution was chosen and used to perform digital PCR for each gene. Digital PCR mixtures (10 µl) consisted of 5 µl of 2×Quantitect Multiplex NoROX PCR master mix (Qiagen Inc, Valencia, CA, USA), 0.1 µl of 50 µM of each forward and reverse primer except for *katG* (0.16 µl), 0.04 µl of 50 µM wild type probe, 0.04 µl of 50 µM mutant probe, 2.72 µl nuclease-free water except for *katG* (2.6 µl) and 2 µl of diluted DNA template. Digital PCR was performed in 384 well formats on a ViiA7 (Applied Biosystems, Life Technologies Corporation, Carlsbad, CA, USA) with initial denaturation at 95°C for 10 min, followed by 45 cycles (except for *katG*, 55) denaturation at 95°C for 15 sec, annealing and extension at 63°C for 1 min.

### Digital PCR on TB in sputum

The 1∶1, 10∶1, 100∶1, and 1000∶1 H37Rv:XDR-TB mixtures were diluted 10 fold serially and 1 ml was spiked into 1 ml aliquots of a common TB-negative sputum sample, vortexed, liquefied by adding 2 ml of 100 µg/ml dithiothreitol (DTT), heated at 37°C for 20 min, followed by DNA extraction using Sputum DNA Isolation Kit (Norgen BioTek Corp., Thorold, ON, Canada) per the manufacturer's instructions. DNA from was then diluted 10 fold serially in nuclease free water and subjected to real-time PCR for *rpoB* in 10 replicates in order to calculate the percentage of positive detections at each dilution. The dilution that yielded approximately 50% PCR positive was chosen and for digital PCR.

### Sequencing of loci

The *katG, rpoB*, *gyrA*, and *rrs* loci were amplified using the primers of Campbell et al [13]. Each 25 µl PCR mixture contained 12.5 µl HotStarTaq master mix (Qiagen Inc, Valencia, CA, USA), 0.15 µl of the forward and reverse 50 µM primers, 7.2 µl nuclease free water, and 5 µl of genomic DNA. PCR was performed on a MyCycler (Bio-Rad, Hercules, CA, USA) and included an initial denaturation step at 95°C for 15 min, followed by 35 cycles of denaturation at 95°C for 30 sec, annealing at 60°C for 30 sec, and elongation at 72°C for 30 sec, with a final elongation step at 72°C for 7 min. PCR products were analyzed on 2% agarose-gels, verified PCR products were purified using MinElute^®^ PCR Purification Kit (Qiagen Inc, Valencia, CA, USA). Purified PCR products were measured spectrophotometrically, diluted with nuclease free water, mixed with primers then submitted to GeneWiz (GeneWiz Inc; South Plainfield, NJ, USA) for DNA sequencing and uploaded to GenBank (KC344734-KC344741).

### Antimicrobial agents

Drugs used were isoniazid (INH), rifampin (RIF), ofloxacin (OFX; all from Sigma-Aldrich, St. Louis, MO, USA), amikacin (AMK; MP Biomedicals, Solon, OH, USA). INH, AMK were dissolved in sterile distilled water. RIF was dissolved in dimethyl sulfoxide. OFX was dissolved in 0.1 N NaOH. All stock solutions were stored in single use aliquots at −80°C.

### Agar proportion method

The drug susceptibility of H37Rv, XDR-TB and the population of H37Rv and XDR-TB in each mixture was tested in Middlebrook 7H10 agar (Difco) by standard procedure with slight modifications [14]. Briefly, 0.5 McFarland of purified H37Rv, XDR-TB and each H37Rv:XDR-TB mixture were diluted 10-fold serially in sterile distilled water and 10 µl of dilutions of 10^−2^ and 10^−3^ were spread onto M7H10 with and without drug and incubated at 37°C. Critical concentrations endorsed by CLSI were used [14]. Results were read 21 days after inoculation of media. At the critical concentration of each drug bacterial growth was counted to enumerate the % of resistant colonies, whereby >1% is defined as resistance.

## Results

### Specificity of probes

**Figure 1:**
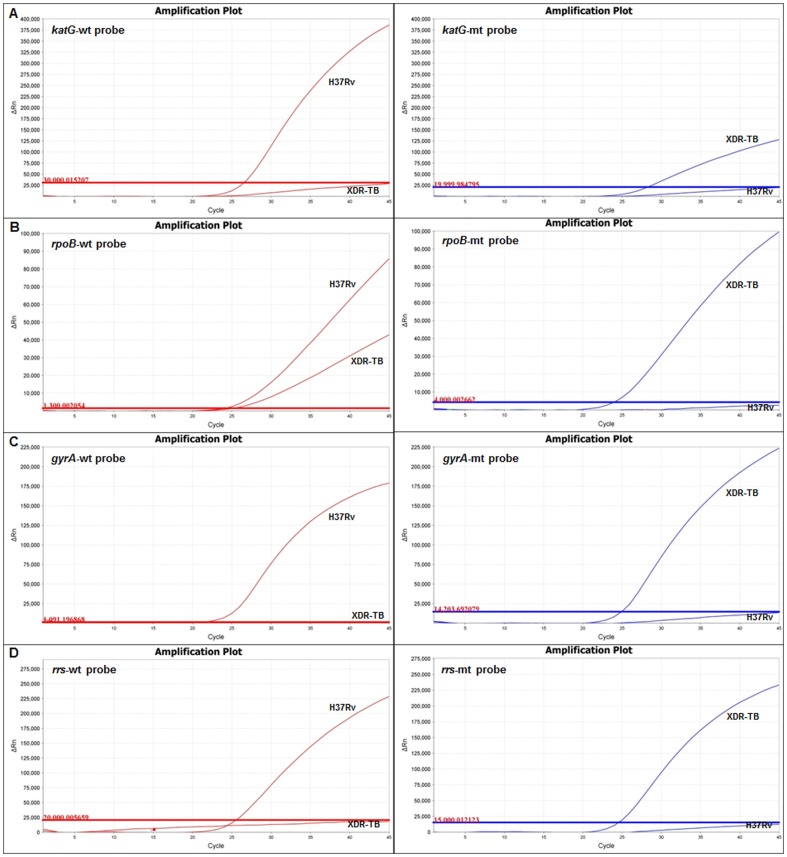
Specificity of real-time PCR probes. Each probe was evaluated with singleplex PCR using purified wild-type (H37Rv) or mutant (XDR-TB) DNA template. Wild-type probe is shown in red, mutant probe in blue. Thresholds for positivity are shown as horizontal lines. All probes were highly specific for cognate DNA sequence, except that the *rpoB* wild-type probe showed significant cross-reactivity. Both *katG* probes, both *rrs* probes, the *rpoB* mutant probe, and the *gyrA* mutant probe displayed minimal cross-reactivity with the opposing template.

Primers and probes were utilized to detect wild-type and mutant sequence for each gene target based on the sequence of the XDR-TB isolate that was used (Table 1). This isolate was chosen because it harbored the Ser531Leu of *rpoB*, Ser315Thr of *katG*, A1401G of *rrs*, and Asp94Gly and Ser95Thr mutations of *gyrA* gene, the most common mutations reported for rifampin, isoniazid, injectables, and quinolones, respectively [13]. Specificity of each probe was stringently tested by singleplex PCR using high-concentration wild-type (H37Rv) and mutant (XDR-TB) DNA template (Fig. 1), amounting to approximately 28,600 cfu Tb cells/PCR reaction (Table S1). The *gyrA* wild-type probe showed high specificity for its target sequence, while *gyrA* mutant, *rpoB* mutant, both *katG* probes, and both *rrs* probes exhibited slight cross reactivity which was manageable with threshold adjustment. The *rpoB* wild-type probe showed substantial cross reactivity to mutant target (Fig.1B).

### Real-time PCR

**Figure 2:**
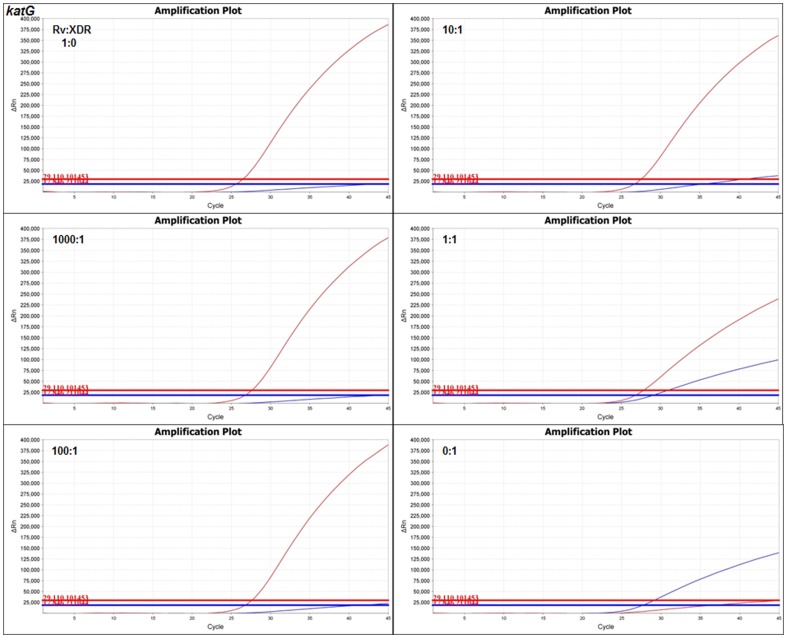
Real-time PCR of undiluted DNA. The undiluted DNA of each H37Rv:XDR-TB mixture underwent real-time PCR for each gene with both probes. *katG* is shown, with detection of mutant sequence (blue trace) at H37Rv:XDR-TB mixtures of 0∶1, 1∶1, and late detection at 10∶1. Detection of wild-type sequence occurred at all H37Rv containing mixtures. qPCR thresholds were set per Figure 1 (red and blue horizontal lines for wild-type and mutant fluorophores, respectively). Similar results were found for other genes (see Figure S1 for *rpoB*, *gyrA*, *rrs*).

The DNA of each H37Rv:XDR-TB mixture, as well as wild type (1∶0) and mutant (0∶1) controls, were amplified by real-time PCR for each gene individually with primers and both probes, using the thresholds of Figure 1. Real-time PCR detected mutant sequence at a 1∶1 mixture of H37Rv:XDR-TB for all four genes (Fig. 2 shows *katG* as an example, for other genes see Figure S1). At a 10∶1 mixture, mutants were weakly detected for all four genes. At 100∶1 and 1000∶1 mixtures, mutant sequence was not detected for any gene (Figure 2, Figure S1).

### Sanger Sequencing

**Figure 3:**
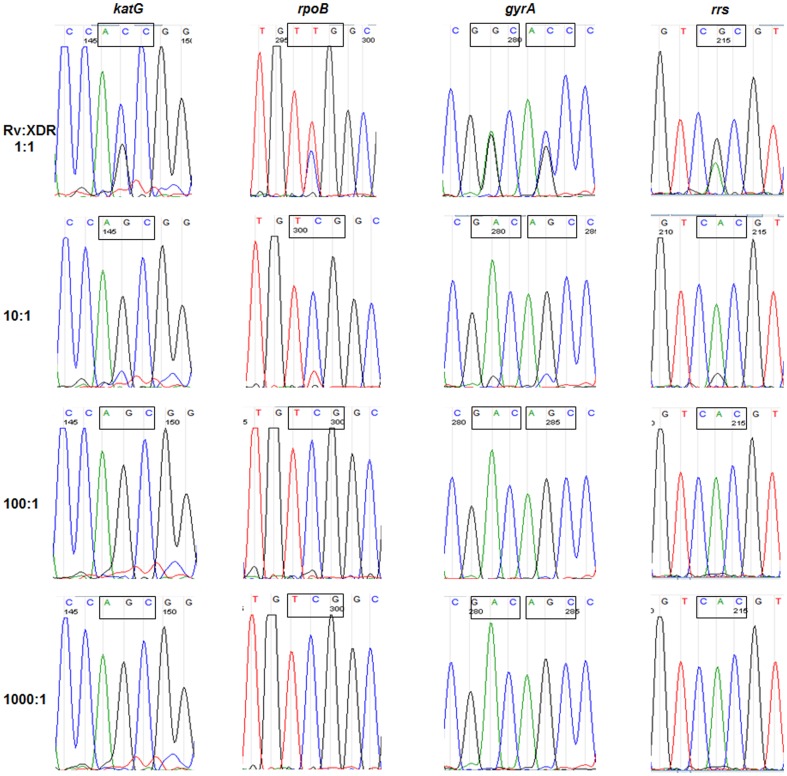
Sanger sequencing of the H37Rv:XDR-TB mixtures. Undiluted DNA underwent PCR and purified products were sequenced. Mixtures of 10∶1, 100∶1, and 1000∶1 were reported as wild-type per the sequencing instrument.

The DNA of each H37Rv:XDR-TB mixture was amplified with locus-specific primers and submitted for sequencing. Traces are shown in Figure 3. Mutant sequence was reported only at the 1∶1 mixture. Subtle peaks of mutant nucleotide were visible at a 10∶1 mixture. The 10∶1, 100∶1, and 1000∶1 mixtures were all reported as wild-type.

### Digital PCR

**Figure 4:**
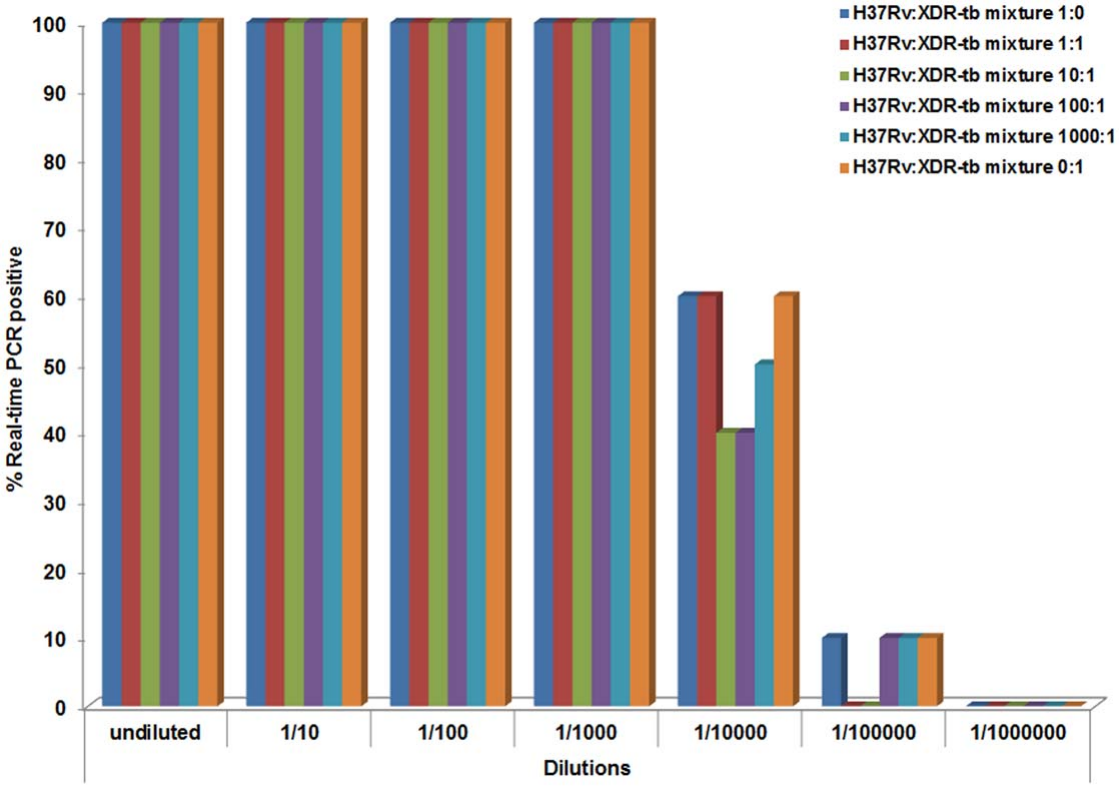
Determination of dilution for digital PCR. The DNA of each H37Rv:XDR-TB mixture was diluted 10 fold serially and subjected to real-time PCR using the *rpoB* primers and probes. The percent of PCR reactions that amplified at each dilution is shown. The target one-half genome equivalent DNA is the amount of DNA that yielded 50% positive [10].

**Figure 5:**
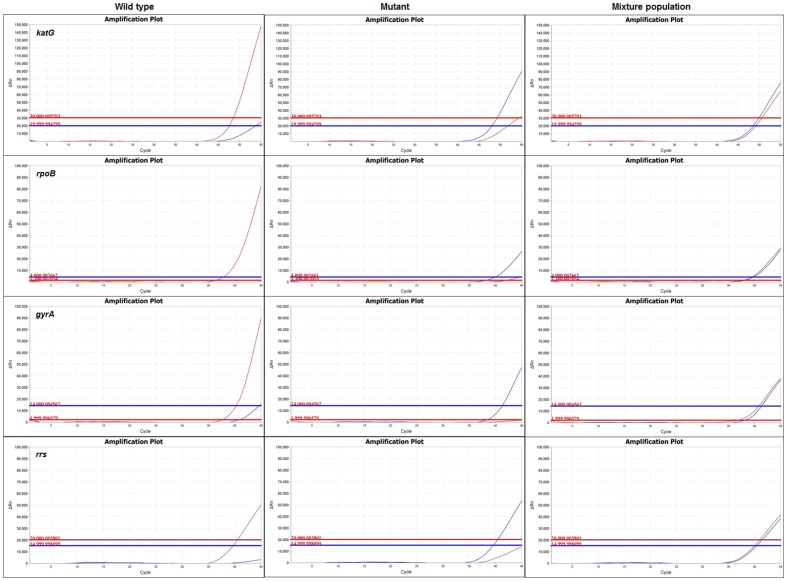
Digital PCR. The DNA of each H37Rv:XDR-TB mixture was diluted 1∶10000 and subjected to real-time PCR for each gene with both probes in 384 well plates. This figure shows representative 10∶1 (*katG, gyrA, rrs*) or 100∶1 (*rpoB*) mixtures. Individual wells revealed detection with the wild-type probe (red traces), mutant probe (blue traces), both, or neither. qPCR thresholds were set per Figure 1 (red and blue horizontal lines for wild-type and mutant fluorophores, respectively). Cycling continued for 55 cycles (for *katG*) due to dilute DNA template.

The DNA of each H37Rv:XDR-TB mixture was then diluted 10 fold serially and subjected to real-time PCR using the *rpoB* primers and probes. The starting number of cells and input DNA are shown in Table S1. The percent of PCR reactions that amplified at each dilution was enumerated and shown in Figure 4. Specifically, at the 1/10000 dilutions approximately 40–60% of PCR reactions were positive for amplification, thus we chose this dilution for digital PCR as previously described [10]. To perform digital PCR the diluted DNA underwent PCR in 384 well plates for each gene individually with primers and both probes. This yielded wild-type, mutant, mixed, and negative results as indicated in Table 2 (see example traces on Figure 5). Positive wells were counted and the percentages of wild-type and mutant templates were calculated (% mutant = [(n mixed population/2)+n mutant]/total n amplified). All mixtures were simultaneously cultured by agar proportion method and percentage of resistant colonies enumerated for comparison. The % of mutant sequence detected by digital PCR mirrored the % of resistant colonies by agar proportion tightly (Table 3). Digital PCR detected mutant DNA (*rpoB* and *rrs*) even at a 1000∶1 mixture of H37Rv:XDR TB. By contrast, real-time PCR or PCR with Sanger sequencing yielded only binary results (‘Resistant’ vs. ‘Susceptible’), and only at lower sensitivities.

**Table 2:** Digital PCR.

Gene	H37Rv: XDR-TB mixture	Number of Reactions	Number with no amplification	Number with wild-type sequence detected only	Number with both wild-type and mutant sequences detected	Number with mutant sequence detected only	% Mutant a
*katG*	1∶0	384	39	345	0	0	0
	1∶1	384	33	81	262	8	39.6
	10∶1	384	99	243	25	17	10.4
	100∶1	384	115	260	6	3	2.2
	1000∶1	384	156	228	0	0	0.0
	0∶1	384	42	1	0	341	99.7
*rpoB* b	1∶0	384	64	320	0	0	0
	1∶1	384	25	75	284	0	39.6
	10∶1	384	106	222	56	0	10.1
	100∶1	384	130	250	2	2	1.2
	1000∶1	384	202	181	0	1	0.5
	0∶1	384	35	1	2	346	99.4
*gyrA*	1∶0	384	82	302	0	0	0
	1∶1	384	60	82	136	106	53.7
	10∶1	384	131	217	16	20	11.1
	100∶1	384	177	206	1	0	0.2
	1000∶1	384	162	222	0	0	0.0
	0∶1	384	69	0	1	314	99.8
*rrs*	1∶0	384	70	314	0	0	0
	1∶1	384	39	59	195	91	54.6
	10∶1	384	45	270	65	4	10.8
	100∶1	384	42	334	8	0	1.2
	1000∶1	384	67	316	1	0	0.2
	0∶1	384	53	0	1	330	99.8

a % mutant = [(n mixed population/2)+n mutant]/total n amplified.

b Since the *rpoB* wild-type probe showed substantial cross reactivity to mutant target (Fig.1B), detection of both wild-type and mutant *rpoB* probes in a single well was interpreted as presence of mutant sequence if fluorescence signal of mutant probe was higher than that of the wild-type probe, and interpreted as a mixed population if fluorescence signal of the mutant probe was equal or lower than that of the wild-type probe. Detection of wild-type only *rpoB* was interpreted as wild-type sequence.

**Table 3:** Comparison of digital PCR, agar proportion, real-time PCR, and PCR with Sanger sequencing.

Gene	H37Rv: XDR-TB mixture	Agar proportion (% Resistant colonies)	Real-time PCR	PCR with Sequencing (Sanger)	Digital PCR (% Resistant colonies)
*katG*	1∶1	R (60.2%)	R	R	R (39.6%)
	10∶1	R (13.8%)	R	S	R (10.4%)
	100∶1	R (3.9%)	S	S	R (2.2%)
	1000∶1	S (0.1%)	S	S	S (0.0%)
*rpoB*	1∶1	R (29.4%)	R	R	R (39.6%)
	10∶1	R (6.9%)	R	S	R (10.1%)
	100∶1	R (2.8%)	S	S	R (1.2%)
	1000∶1	S (0.1%)	S	S	S (0.5%)
*gyrA*	1∶1	R (28.3%)	R	R	R (53.7%)
	10∶1	R (2.2%)	R	S	R (11.1%)
	100∶1	S (0.5%)	S	S	S (0.2%)
	1000∶1	S (0.0%)	S	S	S (0.0%)
*rrs*	1∶1	R (62.1%)	R	R	R (54.6%)
	10∶1	R (7.9%)	R	S	R (10.8%)
	100∶1	R (2.7%)	S	S	R (1.2%)
	1000∶1	S (0.0%)	S	S	S (0.2%)
Correlation with agar proportion			81% (13/16)	56% (9/16)	100% (16/16)

R = resistant. S = susceptible. Agar proportion resistance defined as >1% resistant colonies, and the actual percentage of resistant colonies is shown in parentheses. Digital PCR resistance defined as >1% of PCR reactions indicating mutant sequence, with the actual percentage of PCR reactions with mutant sequence shown in parentheses. Real-time PCR resistance defined as mixed populations of wild-type and mutant sequence. Sequencing defined resistance as evidence of mutant sequence by chromatogram.

### Digital PCR on TB in sputum

To mimic clinical specimens, the H37Rv:XDR-TB mixtures and their dilutions were spiked into sputum. Sputum underwent DNA extraction and this was diluted 10 fold serially to determine the appropriate dilution for digital PCR (approximately 1000 cfu/ml). Digital PCR was performed, and these results yielded a reasonable representation of the H37Rv:XDR-TB mixture, with an average of 74.7±1.7,22.8±2.1, 2.1±1.5, and 0.5±0.3% mutant sequence in spiked H37Rv:XDR-TB mixtures of 1∶1, 10∶1, 100∶1, and 1000∶1 respectively (Table 4).

**Table 4:** Digital PCR of spiked sputum samples using *rpoB* a primers and probes.

H37Rv: XDR-TB mixture	Starting number of Tb cell in spiked sputum (cfu/ml)	Appropriate DNA dilution^b^	Number of Reactions	Number with no amplification	Number with wild-type sequence detected only	Number with both wild-type and mutant sequences detected	Number with mutant sequence detected only	% Mutant^c^
1∶1	1.88×10^6^	1/1000	384	71	40	93	180	72.4
10∶1	1.03×10^6^	1/1000	384	235	107	14	28	23.5
100∶1	0.95×10^6^	1/1000	384	282	99	0	3	2.9
1000∶1	0.94×10^6^	1/1000	384	310	73	1	0	0.7
1∶1	1.88×10^5^	1/100	384	111	38	53	182	76.4
10∶1	1.03×10^5^	1/100	384	237	110	8	29	22.4
100∶1	0.95×10^5^	1/100	384	284	98	0	2	2.0
1000∶1	0.94×10^5^	1/100	384	295	88	1	0	0.6
1∶1	1.88×10^4^	1/10	320	112	35	34	139	75.0
10∶1	1.03×10^4^	1/10	320	213	83	5	19	20.1
100∶1	0.95×10^4^	1/10	320	228	88	2	2	3.3
1000∶1	0.94×10^4^	1/10	320	235	84	1	0	0.6
1∶1	1.88×10^3^	undiluted	27	3	3	6	15	75.0
10∶1	1.03×10^3^	undiluted	27	11	12	0	4	25.0
100∶1	0.95×10^3^	undiluted	27	21	6	0	0	0.0
1000∶1	0.94×10^3^	undiluted	27	23	4	0	0	0.0

a Since the *rpoB* wild-type probe showed substantial cross reactivity to mutant target (Fig.1B), detection of both wild-type and mutant *rpoB* probes in a single well was interpreted as presence of mutant sequence if fluorescence signal of mutant probe higher than that of the wild-type probe, and interpreted as a mixed population if fluorescence signal of the mutant probe was equal or lower than that of wild-type probe. Detection of wild-type only *rpoB* was interpreted as wild-type sequence.

b the dilution that yielded approximately 50% PCR positive.

c % mutant = [(n mixed population/2)+n mutant]/total n amplified.

## Discussion

This work demonstrates the principal of digital PCR for both detection and quantitation of heteroresistance in drug resistant tuberculosis. This method offered an extremely accurate representation of the percentage of resistant colonies that were found by agar proportion in a much shorter timeframe (1 day versus 21), and yielded sensitivity down to as little as 0.1% mutant DNA in the mixture. This speed alone may yield clinical value, particularly as there are instances when a Tb isolate does not grow well during susceptibility testing.

The work urges caution with interpretation of many PCR-based genotypic resistance tests, as is used in line probe, Xpert MTB/RIF, and sequencing methodologies. While these methods offer significant advantages in terms of speed and simplicity of interpretation, we found that qPCR-based probe methods (on DNA from an entire Tb isolate) could detect resistance only when present at a high amount (approximately a 10% mixture). Sanger sequencing was only capable of detecting resistance at a 50/50 mixture (28–60% resistant colonies). Additionally, we examined a laboratory-developed rapid phenotypic DST assay that utilizes qPCR of mycobacteriophage [15] and that method detected resistance at the ∼10% level as well (data not shown).

Our primary purpose of this work was to detect heteroresistance in isolates; however the digital PCR method also worked directly on sputum with sufficient Tb quantity (∼10^3^ cfu Tb/ml sputum, Table 4). We also tested the system on nontuberculous mycobacteria and there was little positivity with most primers and probes across most NTM species (details in Table S2). This is not surprising, since the resistance determining region of *rpoB* is outside the variable region used for detecting other *Mycobacterium* species [16]. However given some cross-reactivity with other species one should use caution if using the assays on a mixed mycobacterial isolate or specimen.

The question, of course, is what is the clinical significance of low-level resistance and how common is the phenomena. Data on clinical significance is limited in this area and needs further study, particularly for second line drugs. However some studies in MDR-TB endemic settings have found heteroresistance to be common, on the order of 30% across several genes [6]. Therefore this is an area of great relevance given increases in MDR-TB globally, the proliferation of PCR-based genotypic assays in the clinical setting, and the potential risk of reporting false susceptibility with these methods should low-level resistance be present.

This was a study to prove the principal of digital PCR, and as such there were limitations. We used a single XDR strain with the most common *katG*315, *rpoB*531, *gyrA*94,95, and *rrs*1401 mutations, thus this assay would function on a Tb isolate with heteroresistance due to of any of these mutations, but does not interrogate others. We chose our XDR strain because it harbored mutations at these key areas, but there are a diversity of other areas [13] that would need to be probed should one wish to devise a broadly applicable clinical diagnostic. Future work includes broadening the assay to incorporate more mutations and evaluating the performance of the assay on incoming clinical isolates against a range of conventional susceptibilities and against patient outcome. As commercial digital PCR platforms are becoming more available, we think this method offers great promise for quantification of drug resistant Tb.

## Supporting Information

Figure S1 **Real-time PCR of undiluted DNA.** The undiluted DNA of each H37Rv:XDR-TB mixture underwent real-time PCR for each gene with both probes. *rpoB, gyrA*, and *rrs* are shown, with detection of mutant sequence (blue trace) at various H37Rv:XDR-TB mixtures. qPCR thresholds were set per Figure 1 (red and blue horizontal lines for wild-type and mutant fluorophores, respectively).
(TIF)

Table S1 **Starting number and amount of cells in each PCR well for each diluted DNA template.**
(DOC)

Table S2 **Specificity of each probe versus non-tuberculous mycobacteria (NTM).**
(DOCX)
