# Supplementary figures and images for: Digital PCR to Detect and Quantify Heteroresistance in Drug Resistant Mycobacterium tuberculosis
Source: PLoS One. 2013 Feb 27;8(2):e57238. doi: 10.1371/journal.pone.0057238 (PMC3584134; doi:10.1371/journal.pone.0057238)

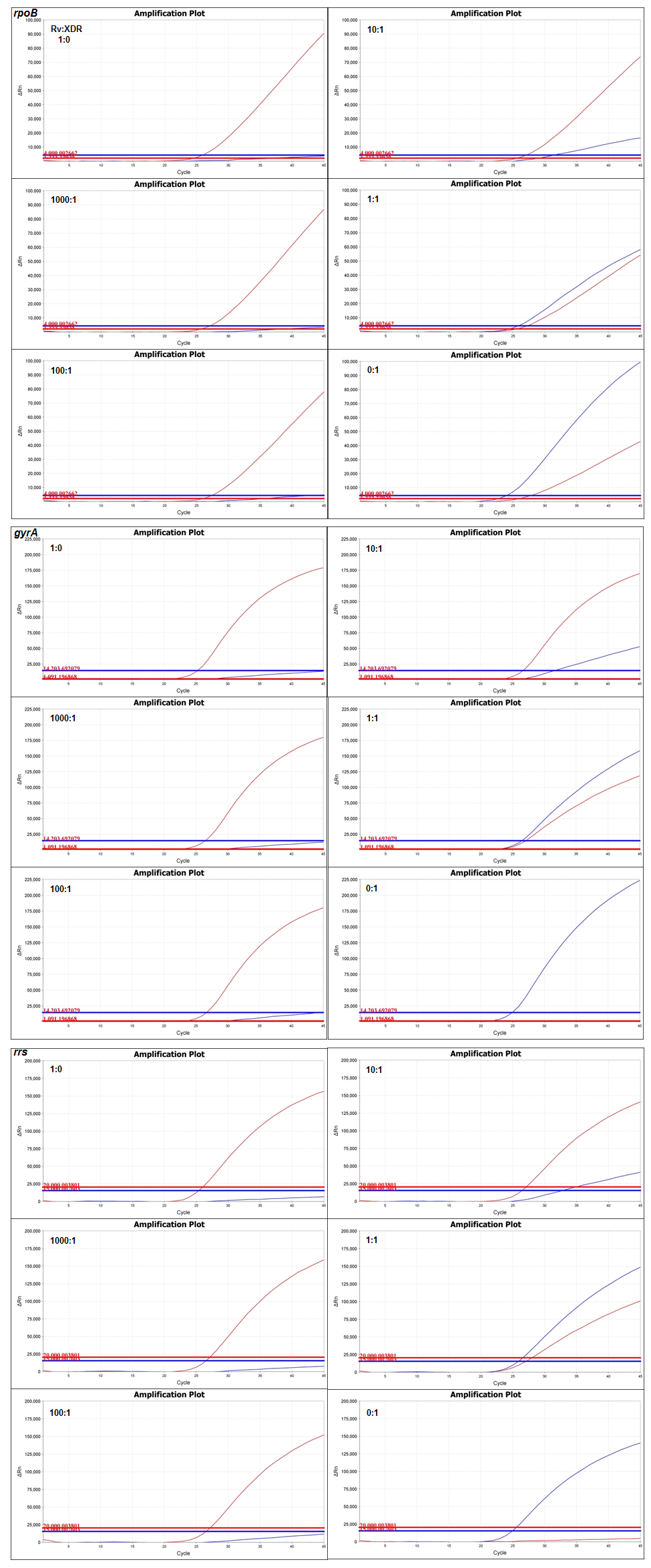

Supplement: Figure S1 — Real-time PCR of undiluted DNA. The undiluted DNA of each H37Rv:XDR-TB mixture underwent real-time PCR for each gene with both probes. rpoB, gyrA, and rrs are shown, with detection of mutant sequence (blue trace) at various H37Rv:XDR-TB mixtures. qPCR thresholds were set per Figure 1 (red and blue horizontal lines for wild-type and mutant fluorophores, respectively). (TIF) [file pone.0057238.s001.tif]
