# Supplementary material for: Digital PCR to Detect and Quantify Heteroresistance in Drug Resistant Mycobacterium tuberculosis
Source: PLoS One. 2013 Feb 27;8(2):e57238. doi: 10.1371/journal.pone.0057238 (PMC3584134; doi:10.1371/journal.pone.0057238)
Supplement: Table S1 — Starting number and amount of cells in each PCR well for each diluted DNA template. (DOC) [file pone.0057238.s002.doc]

Table S1. Starting number and amount of cells in each PCR well for each diluted DNA template.

| H37Rv:XDR-TB mixtures | Starting number of cells | | | | Dilutions | | | | | | | | | | | |
| --- | --- | --- | --- | --- | --- | --- | --- | --- | --- | --- | --- | --- | --- | --- | --- | --- |
|  |  |  |  |  | 1/10 | | 1/100 | | 1/1000 | | 1/10000 | | 1/100000 | | 1/1000000 | |
|  | H37Rv (cfu/ml) | XDR-TB(cfu/ml) | Total (cfu/ml) | Total cfu/PCR reaction* | cfu/ml | cfu/PCR reaction | cfu/ml | cfu/PCR reaction | cfu/ml | cfu/PCR reaction | cfu/ml | cfu/PCR reaction | cfu/ml | cfu/PCR reaction | cfu/ml | cfu/PCR reaction |
| 1:0 | 1.59x10^7^ | 0 | 1.59x10^7^ | 31800 | 1.59x10^6^ | 3180 | 1.59x10^5^ | 318 | 1.59x10^4^ | 31.8 | 1.59x10^3^ | 3.18 | 159 | 0.32 | 15.9 | 0.032 |
| 1:1 | 1.66x10^7^ | 5.98x10^6^ | 2.26x10^7^ | 45200 | 2.26x10^6^ | 4520 | 2.26x10^5^ | 452 | 2.26x10^4^ | 45.2 | 2.26x10^3^ | 4.52 | 226 | 0.45 | 22.6 | 0.045 |
| 10:1 | 1.02x10^7^ | 1.34x10^6^ | 1.15x10^7^ | 23000 | 1.15x10^6^ | 2300 | 1.15x10^5^ | 230 | 1.15x10^4^ | 23 | 1.15x10^3^ | 2.3 | 115 | 0.23 | 11.5 | 0.023 |
| 100:1 | 1.16x10^7^ | 4.7x10^5^ | 1.21x10^7^ | 24200 | 1.21x10^6^ | 2420 | 1.21x10^5^ | 242 | 1.21x10^4^ | 24.2 | 1.21x10^3^ | 2.42 | 121 | 0.24 | 12.1 | 0.024 |
| 1000:1 | 1.08x10^7^ | 6.0x10^3^ | 1.09x10^7^ | 21800 | 1.09x10^6^ | 2180 | 1.09x10^5^ | 218 | 1.09x10^4^ | 21.8 | 1.09x10^3^ | 2.18 | 109 | 0.22 | 10.9 | 0.022 |
| 0:1 | 0 | 1.28x10^7^ | 1.28x10^7^ | 25600 | 1.28x10^6^ | 2560 | 1.28x10^5^ | 256 | 1.28x10^4^ | 25.6 | 1.28x10^3^ | 2.56 | 128 | 0.26 | 12.8 | 0.026 |
| Average | | | 1.43x10^7^ | 28600 | 1.43x10^6^ | 2860 | 1.43x10^5^ | 286 | 1.43x10^4^ | 28.6 | 1.43x10^3^ | 2.86 | 143 | 0.29 | 14.3 | 0.029 |

*Each PCR reaction contained 2 μl of DNA template
