# Supplementary material for: Digital PCR to Detect and Quantify Heteroresistance in Drug Resistant Mycobacterium tuberculosis
Source: PLoS One. 2013 Feb 27;8(2):e57238. doi: 10.1371/journal.pone.0057238 (PMC3584134; doi:10.1371/journal.pone.0057238)
Supplement: Table S2 — Specificity of each probe versus non-tuberculous mycobacteria (NTM). (DOCX) [file pone.0057238.s003.docx]

Table S2. Specificity of each probe versus non-tuberculous mycobacteria (NTM)

| Species | Probes | | | | | | | |
| --- | --- | --- | --- | --- | --- | --- | --- | --- |
|  | *rpoB* | | *katG* | | *gyrA* | | *rrs* | |
|  | wild-type | mutant | wild-type | mutant | wild-type | mutant | wild-type | mutant |
| *M. avium* ATCC 700 | No detection | No detection | No detection | No detection | No detection | No detection | cross-reactivity | No detection |
| *M. intracellulare* ATCC 13950 | No detection | No detection | No detection | No detection | No detection | No detection | cross-reactivity | No detection |
| *M. simiae* ATCC 25275 | No detection | No detection | No detection | No detection | No detection | No detection | No detection | No detection |
| *M. kansasii* ATCC 12478 | No detection | No detection | No detection | No detection | No detection | No detection | cross-reactivity | No detection |
| *M. sherisii* S53 | cross-reactivity | No detection | No detection | No detection | No detection | No detection | No detection | No detection |
| *M. fortuitum* ATCC 6841 | cross-reactivity | No detection | No detection | No detection | cross-reactivity | cross-reactivity | cross-reactivity | No detection |
| *M. peregrinum* ATCC 700686 | cross-reactivity | No detection | No detection | No detection | No detection | No detection | cross-reactivity | No detection |
